# Ability exposure in physical education and university students’ psychological well-being: evaluation anxiety as a mediator and PE belonging and disadvantaged status as contextual conditions

**DOI:** 10.3389/fpsyg.2026.1839395

**Published:** 2026-06-17

**Authors:** Haibo Wang

**Affiliations:** School of Physical Education, Hechi University, Hechi, China

**Keywords:** ability exposure, disadvantaged status, evaluation anxiety, PE belonging, physical education, psychological well-being

## Abstract

Physical education (PE) is often regarded as beneficial for students’ physical and psychosocial development; however, its psychological correlates may depend on the evaluative conditions under which participation occurs. Drawing on the view that PE is an embodied, publicly performed, and socially comparable context, this cross-sectional study examined whether ability exposure in PE was associated with university students’ psychological well-being through evaluation anxiety, and whether PE belonging and disadvantaged status functioned as contextual conditions. A survey was completed by 648 undergraduate students enrolled in PE courses. Results showed that ability exposure was associated with higher evaluation anxiety and lower psychological well-being. Evaluation anxiety statistically mediated the negative association between ability exposure and psychological well-being. PE belonging was associated with lower evaluation anxiety and higher psychological well-being, whereas disadvantaged status was associated with higher evaluation anxiety and lower psychological well-being. However, the hypothesized moderation effects and the indices of moderated mediation were not statistically significant. Given the cross-sectional correlational design, these findings should be interpreted as associations rather than causal effects. The study suggests that the psychological meaning of PE may depend less on participation itself than on how bodily performance is made visible, comparable, and open to judgment.

## 1 Introduction

Physical education (PE) is widely recognized as an important component of students’ holistic development, contributing not only to physical health but also to social interaction, emotional growth, and school engagement (Ryan and Deci, 2000). In higher education, PE can provide opportunities for movement, cooperation, self-expression, and the development of healthy lifestyles during a formative stage of identity development, thereby shaping students’ broader psychosocial functioning (Han et al., 2025). Yet PE differs from many other university learning contexts in a crucial respect: it is an embodied, publicly performed, and socially comparable environment. Students do not participate in PE only through ideas or verbal responses; they participate through their bodies, movements, coordination, and visible competence. As a result, PE is not merely a course about exercise, but also a social setting in which bodily ability is displayed, observed, compared, and sometimes informally ranked. Although existing research has largely emphasized the developmental and health-promoting benefits of PE, it has paid comparatively less attention to the evaluative pressure and emotional risk embedded in PE as a public form of bodily practice (Leisterer and Jekauc, 2019; Andresen et al., 2023).

This neglected dimension matters because the psychological meaning of PE may depend less on participation per se than on the conditions under which participation occurs. PE is often assumed to be inherently beneficial because it involves movement and engagement; however, its emotional consequences are likely to be conditional rather than uniformly positive. When participation takes place under conditions of public visibility, when ability differences become salient, when mistakes can be witnessed by peers, and when bodily performance becomes socially readable, PE may generate not only enjoyment and confidence but also anxiety, embarrassment, and self-threat. In this sense, PE may simultaneously function as a developmental resource and an emotionally risky context. This conditional perspective is particularly important in university settings, where peer evaluation, self-presentation, and competence-related identity concerns may be especially salient (Baumeister and Leary, 1995; Gopalan et al., 2022; Dost and Mazzoli Smith, 2023). A fuller understanding of PE and student well-being therefore requires attention to the social and evaluative architecture of embodied participation, rather than assuming that exposure to movement-based education is automatically beneficial.

Against this background, the present study focuses on ability exposure as a key feature of PE experience. Ability exposure does not simply refer to general classroom participation; rather, it refers to the extent to which students perceive their bodily performance, movement competence, and physical shortcomings as publicly visible and open to comparison or judgment in PE classes. The central argument of this study is that the psychological cost of such exposure is unlikely to arise merely from being seen, but from being seen under conditions that imply evaluation. In PE, bodily visibility can become psychologically consequential when students anticipate that their performance may be judged by teachers or peers, interpreted as evidence of competence or inadequacy, and used as a basis for social comparison. This argument is consistent with broader work on social-evaluative threat, which suggests that stress reactions are especially pronounced when individuals are exposed to situations in which the self may be judged negatively by others (Dickerson and Kemeny, 2004). It also resonates with research showing that perceived athletic competence in PE is meaningfully linked to fear of negative evaluation, indicating that visible performance can become a psychologically charged experience rather than a neutral pedagogical requirement (Ridgers et al., 2007). For this reason, the present study proposes that evaluation anxiety constitutes a central psychological mechanism linking ability exposure to psychological well-being. Put differently, what may matter most is not simply whether ability differences exist, but how those differences are publicized, socialized, and evaluatively experienced within the classroom. From this perspective, the pathway of interest is not merely exposure and outcome, but a more specific process in which visibility fosters evaluative anxiety, which in turn undermines well-being.

This focus on evaluation anxiety is important because the construct captures more than general nervousness or routine performance concern. Rather, it reflects the tension, self-consciousness, and anticipatory unease that arise when individuals believe they may be judged, found inadequate, or negatively appraised by others (Leary, 1983). In PE, such anxiety may be especially salient because bodily performance unfolds immediately and publicly, making mistakes easier to witness and competence differences harder to conceal. The psychological burden of PE may therefore arise not merely from performing, but from performing under conditions of anticipated judgment. When such evaluative concern becomes repeated or chronic, it may erode positive affect, emotional stability, self-acceptance, and broader psychological well-being (Watson et al., 1988; Ryff and Keyes, 1995). Conceptualizing evaluation anxiety as the mediating process thus helps explain why PE can be experienced as emotionally beneficial in some circumstances yet psychologically taxing in others.

At the same time, students do not experience PE under identical social conditions. The emotional consequences of ability exposure may also be shaped by the broader relational and positional contexts in which participation takes place. In particular, PE belonging may serve as an important interpersonal resource, reflecting the extent to which students feel accepted, respected, and socially anchored in PE classes. A strong sense of belonging is widely understood as a basic psychological condition that supports emotional security, motivation, and adjustment (Baumeister and Leary, 1995; Ryan and Deci, 2000), and in higher education it has been closely linked to students’ mental health and overall well-being (Gopalan et al., 2022; Dost and Mazzoli Smith, 2023). Likewise, students who perceive themselves as occupying a disadvantaged position in PE may enter the classroom with greater vulnerability, lower confidence, or heightened sensitivity to evaluation. These contextual conditions may shape how threatening PE feels overall and may also influence the strength of the focal psychological process. However, rather than assuming in advance that they must function as strong moderators, it may be more theoretically appropriate to consider PE belonging and disadvantaged status as broader protective and risk conditions that structure students’ emotional ecology in PE. This framing allows for a more nuanced account in which relational safety and positional vulnerability matter, while leaving open the question of whether they substantially alter the core mechanism linking exposure, anxiety, and well-being.

Accordingly, the present study develops and tests a model linking ability exposure, evaluation anxiety, PE belonging, disadvantaged status, and psychological well-being among university students. Relative to broad models of well-being in the extant literature, the present framework is narrower and more situational: rather than attempting to explain psychological well-being in its entirety, it isolates one PE-specific pathway through which public bodily visibility may become emotionally consequential. At the same time, the model differs from performance-oriented models of PE that emphasize motor competence, achievement, or observable behavioral outcomes. Here, the central concern is not whether students perform better or worse in PE, but how they psychologically experience participating under conditions of visibility, comparison, and anticipated judgment. In that sense, the study contributes to the literature by integrating a contextualized well-being mechanism with inclusion-oriented PE scholarship while keeping performance-relevant self-perceptions in view as covariates rather than as the focal explanatory pathway.

## 2 Literature review

### 2.1 Physical education as an evaluative embodied context: from participation to public exposure

Physical education (PE) has long been regarded as a valuable educational context for supporting students’ physical health, social interaction, and psychosocial development. A substantial body of research has highlighted its positive contributions to physical fitness, cooperation, peer connectedness, emotional adjustment, and the cultivation of healthy lifestyles (Jansson et al., 2021; Vazou et al., 2017). Within this dominant perspective, PE is often understood as a setting in which students build motor competence, develop teamwork, and benefit from movement-based participation in ways that support both individual growth and social inclusion. This positive narrative has been especially influential in studies linking PE to school engagement, inclusion, and well-being, where participation itself is frequently treated as beneficial. However, such an account may overlook a defining feature of PE that distinguishes it from many other educational settings: PE is not simply participatory, but deeply embodied, publicly performed, and socially comparable (Åsebø et al., 2021; Tudor et al., 2018). Students do not engage in PE only through verbal responses, written outputs, or abstract cognitive performance; rather, they engage through visible bodily action, movement coordination, physical skill, and observable competence. As a result, PE is not merely a course about exercise, but also a social setting in which bodily performance can be displayed, witnessed, compared, and informally ranked. In many PE classrooms, students are required to demonstrate skills, perform in front of others, participate in visibly comparative activities, respond to corrective feedback publicly, or make mistakes that are immediately observable to peers and teachers. Even when formal assessment is not strongly emphasized, PE often contains an implicit evaluative structure in which bodily differences become socially legible (Metz et al., 2024; Åsebø et al., 2021). The interactive and dynamic qualities that make PE educationally rich may therefore also make it emotionally complex, because they render students’ physical abilities visible in ways that are difficult to hide, delay, or reinterpret. Research on PE-related stressors has similarly shown that exposure, comparison, and the possibility of public failure are often built into students’ everyday classroom experience rather than appearing only in formal testing situations (Tudor et al., 2018).

For this reason, participation in PE cannot be assumed to be psychologically neutral. The public and embodied nature of PE means that bodily competence is often exposed under conditions of observation, comparison, and potential judgment, making visible not only what students can do but also where they struggle. Against this background, the present study conceptualizes ability exposure as a specific experiential condition of PE rather than as a general form of classroom participation. Ability exposure refers to the extent to which students perceive their bodily performance, movement competence, and physical shortcomings as publicly visible and open to comparison or judgment in PE classes. It involves several interconnected elements, including the public visibility of performance, the salience of social comparison, concern about being judged, and the heightened visibility of one’s weaknesses in front of others. Conceptually, this is important because it shifts the focus from participation as an educational act to exposure as a psychological experience. When students perceive that their performance is highly visible, easily comparable, and potentially interpretable as evidence of competence or inadequacy, PE may become not only a developmental resource but also a setting of evaluative vulnerability. In such contexts, what matters is not simply whether students participate, but whether they participate under conditions that make their bodies socially readable and emotionally risky. This interpretation is consistent with studies showing that social acceptance and perceived motor competence are closely intertwined in PE contexts, such that students’ physical ability is often experienced not only as performance but also as a socially meaningful marker of status and acceptance (de Bruijn and van der Wilt, 2023). It also aligns with evidence that socially evaluative features of PE are associated with anxiety-related responses, including social physique anxiety and fear of failure, particularly when students perceive the classroom climate as judgmental or controlling (Cox et al., 2011; Huéscar Hernández et al., 2020). The more strongly students experience their bodily competence as publicly exposed, the more likely they may be to feel that they are being watched, compared, and potentially judged, thereby heightening concern about how they are seen by others. This suggests that the emotional consequences of PE depend not only on activity itself, but also on the public and evaluative conditions under which activity occurs. Therefore, the present study proposes that ability exposure in PE is positively associated with evaluation anxiety.

### 2.2 Evaluation anxiety as the psychological mechanism linking exposure to well-being

To understand why ability exposure in PE may undermine students’ psychological well-being, it is necessary to move beyond exposure itself and focus on the psychological process through which exposure becomes emotionally consequential. In the present study, this process is conceptualized in terms of evaluation anxiety. Evaluation anxiety is not equivalent to general test anxiety, nor is it simply a form of competitive nervousness associated with sports performance. Rather, it refers to the tension, self-consciousness, and avoidance tendency that arise when individuals anticipate that they may be judged, negatively evaluated, ridiculed, or seen as inadequate by others, a pattern that closely aligns with broader conceptualizations of social anxiety as involving fear, self-focused attention, and avoidance in evaluative interpersonal situations (Morrison and Heimberg, 2013). It is therefore best understood as a form of social-evaluative threat that is activated when performance becomes publicly legible and personally consequential. This distinction is important because students in PE may not be distressed merely because they are active, challenged, or physically engaged, but because bodily action in PE often takes place under conditions that make competence highly visible and socially interpretable. In such circumstances, students may become acutely aware not only of what they are doing, but of how they are being seen while doing it. The psychological cost of PE may arise not merely from performing, but from performing under conditions of anticipated judgment. In this sense, evaluation anxiety represents the subjective experience through which public exposure is translated into emotional strain. When ability becomes visible in a setting where peers and teachers can observe, compare, and infer competence in real time, students may begin to monitor themselves excessively, fear embarrassment, anticipate negative impressions, and hesitate to participate fully. This logic is consistent with research showing that body-related embarrassment is a powerful self-conscious emotion linked to being seen and potentially evaluated by others (Vani et al., 2020). Related work on body- and appearance-based self-conscious emotions further suggests that visible performance can activate shame-, embarrassment-, and inadequacy-related responses when individuals perceive their bodies as objects of social appraisal (Castonguay et al., 2014a,b). In a similar vein, research on fear of evaluation has shown that anxiety may be elicited not only by anticipated criticism, but by the broader prospect of being socially appraised at all, whether that appraisal is explicitly negative or positively valenced but still performance-relevant (Weeks et al., 2008). This makes evaluation anxiety especially relevant to PE, where students’ bodily actions are difficult to conceal, reinterpret, or delay and where public performance may carry implications for competence, status, and social image. Empirical work has also shown that reactivity to socially evaluative stimuli is heightened when individuals are sensitive to judgment, further underscoring the emotional significance of being observed under conditions of potential appraisal (Reichenberger et al., 2019).

This mechanism may be especially salient in PE because bodily performance is more difficult to conceal, reinterpret, or delay than many other forms of classroom performance. Unlike written or verbal tasks, physical execution unfolds immediately and visibly, making errors easier to detect, differences easier to compare, and shortcomings harder to hide. Moreover, bodily competence in PE may carry implications that extend beyond task performance alone, touching on self-worth, social image, peer recognition, and perceived adequacy within the group. Research on the bivalent fear of evaluation model suggests that concerns about how one will be judged can broadly shape self-presentation, withdrawal, and emotional vulnerability in social contexts, which helps explain why evaluative concern in PE may be especially psychologically consequential (Weeks and Howell, 2012). Related evidence further indicates that fear of evaluation is tied to submissive withdrawal and maladaptive cognitive responses, suggesting that students who become preoccupied with how they are seen may be less able to participate confidently and more likely to experience internalized distress (Weeks et al., 2005). Under repeated or persistent conditions of evaluative anxiety, students may experience reduced positive affect, diminished emotional stability, lower self-satisfaction, and weakened vitality, all of which are central components of psychological well-being. This general link between anxiety and diminished well-being has also been supported in educational research showing reciprocal and negative associations between anxiety and subjective well-being (Steinmayr et al., 2016). From this perspective, the impact of ability exposure on well-being is unlikely to be fully direct; rather, it is likely to operate through the anxious anticipation of judgment that visible performance evokes. This makes evaluation anxiety a theoretically plausible and psychologically precise pathway linking exposure to diminished well-being. Accordingly, the present study proposes that evaluation anxiety is negatively associated with psychological well-being and that it mediates the relationship between ability exposure and psychological well-being. In other words, the more students perceive their bodily competence as publicly exposed in PE, the more likely they are to experience evaluation anxiety, and the more such anxiety is activated, the more likely their psychological well-being is to be compromised.

### 2.3 Belonging and disadvantaged status as contextual conditions of PE experience

The psychological experience of PE is shaped not only by how visible students feel, but also by the relational and positional conditions under which that visibility is encountered. In this respect, PE belonging can be understood as an important contextual resource. A sense of belonging in PE refers to the extent to which students feel accepted, respected, supported, and socially anchored in the classroom. More broadly, research has consistently shown that belonging is a foundational condition of students’ adjustment, engagement, and well-being across educational settings (Allen et al., 2018; Furrer and Skinner, 2003). Students who experience stronger belonging are more likely to perceive PE as a space in which they are recognized as legitimate participants rather than as exposed performers under scrutiny. Theoretically, such belonging may reduce the perceived threat associated with public participation by fostering interpersonal safety, lowering self-consciousness, and buffering against the fear of negative judgment. This interpretation is also consistent with intervention-based evidence showing that even relatively brief improvements in social belonging can yield meaningful benefits for students’ academic and health-related outcomes (Walton and Cohen, 2011). In PE specifically, where participation is both visible and socially negotiated, the extent to which students feel accepted by others may be especially consequential. A supportive and inclusive social climate can make public participation feel less threatening, whereas weak belonging may heighten the emotional cost of being seen. In addition, belonging is likely to contribute directly to students’ psychological well-being because feeling included and supported in a socially visible environment may strengthen emotional comfort, self-acceptance, and positive engagement. From this perspective, PE belonging is not merely a pleasant classroom feature, but a condition that may fundamentally shape how students interpret and emotionally respond to PE experiences. At minimum, it should be associated with lower evaluation anxiety and better psychological well-being, even if the precise form of its influence on the core mechanism remains to be determined.

Alongside belonging as a contextual resource, disadvantaged status represents a contextual risk condition. In the present study, this is not treated as a fixed or externally imposed label, but as a self-perceived disadvantaged position in PE. This distinction is conceptually important because students’ emotional responses are likely to depend not only on objective performance differences, but also on whether they subjectively experience themselves as vulnerable, less competent, easily embarrassed, or socially marginalized in PE settings. Research on inclusive PE has repeatedly shown that students who occupy less advantaged positions in movement-based environments may encounter barriers related to participation, recognition, and social acceptance, particularly when classroom practices fail to accommodate difference (Goodwin and Watkinson, 2000; Block and Obrusnikova, 2007). Related work has also shown that PE experiences are shaped by students’ perceived capacity to participate, their functional confidence, and the degree to which teaching practices support or constrain their sense of competence and inclusion (Bertills et al., 2018). Although much of this literature has focused on disability and formally recognized inclusion categories, the broader implication is that disadvantage in PE is not only structural but also experiential: students may feel exposed, out of place, or at risk of embarrassment even in the absence of an externally assigned label. This point is reinforced by scholarship on ableism and educational exclusion, which suggests that vulnerability often emerges through social norms of competence, normality, and legitimate participation rather than through formal status categories alone (Bogart and Dunn, 2019; Page et al., 2021). Students who perceive themselves as occupying a disadvantaged position may therefore enter PE with heightened anticipatory concern, lower emotional security, and a greater expectation of being exposed or negatively judged. Such students may be more likely to experience evaluation anxiety and lower psychological well-being overall. At the same time, PE belonging and disadvantaged status may shape the emotional conditions under which students experience PE, either by conditioning the focal pathways or by functioning as broader protective and risk contexts. That is, belonging may reduce the threat associated with ability exposure, whereas disadvantaged status may intensify the harmful consequences of anxiety; yet, given the complexity of classroom emotional processes, these variables may also operate more diffusely by structuring students’ overall emotional ecology rather than by producing strong and narrowly defined moderation effects. This more cautious formulation is also consistent with broader work on inclusion in PE, which suggests that students’ experiences are shaped by multiple overlapping interpersonal and contextual influences, including peer processes, classroom norms, and the extent to which difference is socially accommodated (Ruscitti et al., 2017; Tarantino et al., 2022; Lawler et al., 2021). Accordingly, the present study proposes that PE belonging is positively associated with psychological well-being and negatively associated with evaluation anxiety, whereas disadvantaged status is negatively associated with psychological well-being and positively associated with evaluation anxiety. In addition, PE belonging and disadvantaged status are expected to shape, to some extent, the strength of the focal relationships among ability exposure, evaluation anxiety, and psychological well-being.

**Figure 1:**
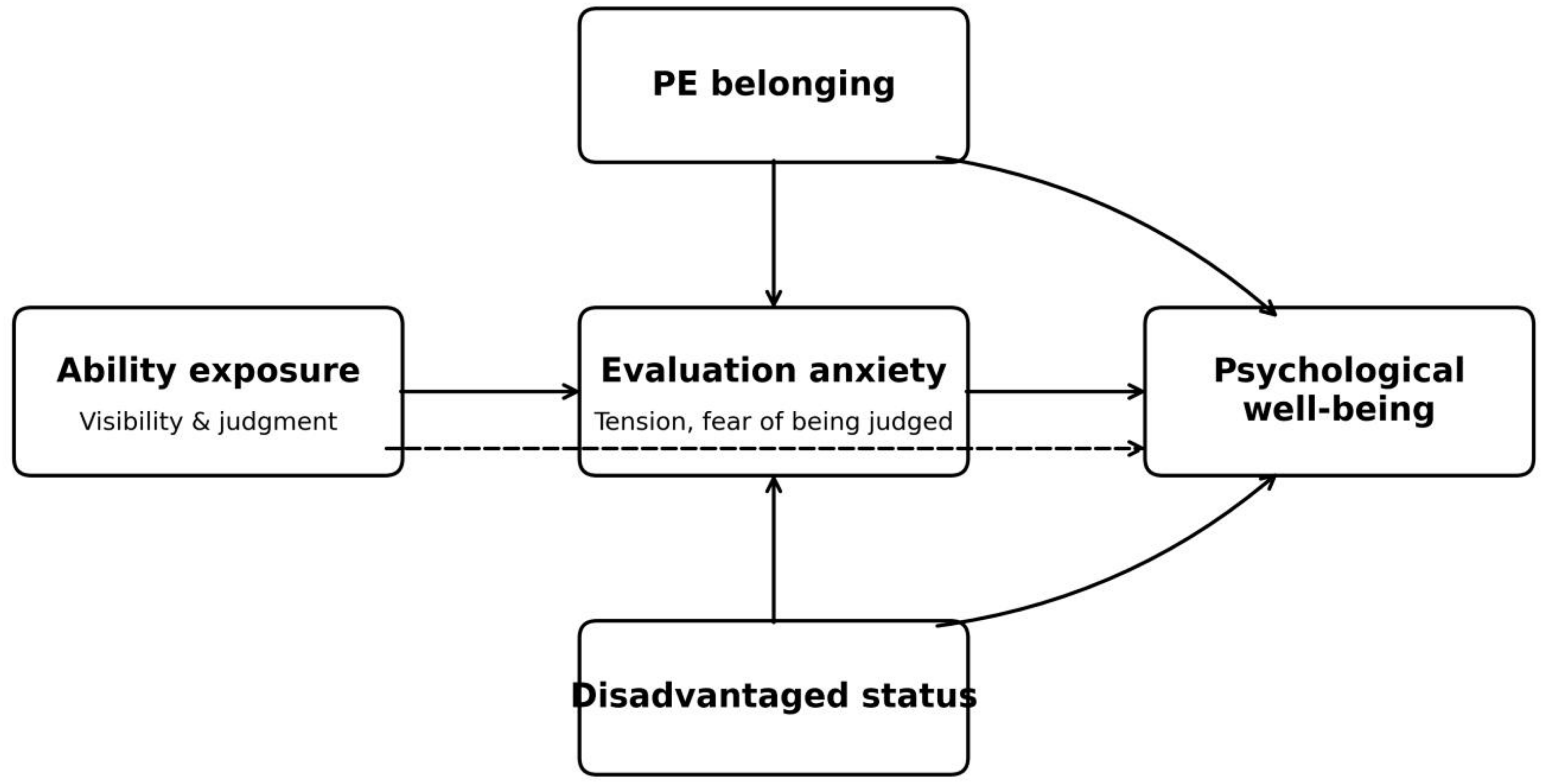
Conceptual model of the study variables.

Building on the foregoing discussion, the present study proposes a conceptual model in which ability exposure in PE is expected to be positively associated with evaluation anxiety, which in turn is expected to be negatively associated with psychological well-being. PE belonging is conceptualized as a contextual protective resource, whereas disadvantaged status is treated as a contextual risk condition. Both are expected to show direct associations with evaluation anxiety and psychological well-being and are examined as possible contextual influences on the focal relationships. Figure 1 presents the proposed conceptual model.

#### 2.3.1 Hypotheses

*H1.* Ability exposure in PE is positively associated with evaluation anxiety.

*H2.* Evaluation anxiety is negatively associated with psychological well-being.

*H3.* Evaluation anxiety mediates the relationship between ability exposure and psychological well-being.

*H4.* PE belonging is negatively associated with evaluation anxiety and positively associated with psychological well-being.

*H5.* Disadvantaged status is positively associated with evaluation anxiety and negatively associated with psychological well-being. In addition, PE belonging and disadvantaged status are examined as supplementary contextual tests of whether the focal relationships among ability exposure, evaluation anxiety, and psychological well-being vary across students' PE experiences.

## 3 Method

### 3.1 Participants and procedure

This study employed a cross-sectional online questionnaire design to examine the relationships among ability exposure in physical education (PE), evaluation anxiety, psychological well-being, PE belonging, and disadvantaged status among university students. The target population consisted of undergraduate students who were currently enrolled in university PE courses, and data were collected from first-, second-, and third-year students who met the study eligibility criteria. The final analytic sample comprised 648 undergraduate students, a size that was adequate for the correlational, mediation, and moderation analyses conducted in the study and that provided stable estimation for the bootstrap-based indirect effects.

Of the 648 participants, 307 (47.4%) were male and 341 (52.6%) were female. In terms of academic year, 203 (31.3%) were freshmen, 227 (35.0%) were sophomores, and 218 (33.6%) were juniors. Regarding major type, 309 (47.7%) were from arts-related disciplines and 339 (52.3%) were from science-related disciplines. With respect to self-perceived athletic competence, 66 (10.2%) students rated themselves as very weak, 120 (18.5%) as relatively weak, 245 (37.8%) as average, 151 (23.3%) as relatively strong, and 66 (10.2%) as very strong. In addition, 173 students (26.7%) identified themselves as being in a disadvantaged position in PE contexts, whereas 475 (73.3%) did not. The sampling frame covered students attending the participating PE classes during survey administration; however, because the anonymized dataset did not retain the total enrollment count for all approached classes, the precise population size and response rate could not be reconstructed retrospectively.

Participants completed an online self-report questionnaire assessing their experiences in PE classes and their recent psychological functioning. The survey link was distributed to eligible students enrolled in university PE courses, and participation was open to those who met the inclusion criteria. Responses were completed independently rather than under direct classroom supervision, and no PE teachers were present during survey completion. The questionnaire included demographic items, items measuring the focal psychosocial constructs, and a self-identification item for disadvantaged status in PE settings. All responses were anonymous, and participation was voluntary. Before completing the questionnaire, participants were informed of the purpose of the study and assured that their responses would be used only for academic research. The analytic dataset contained no missing values on the focal variables. Informed consent was obtained from all participants prior to data collection. Because the data were collected through an online individual-level survey and no class- or teacher-level identifiers were retained, the analyses were conducted at the individual level and did not model classroom clustering. The study was conducted in accordance with the ethical principles of psychological and educational research.

### 3.2 Measures

Unless otherwise specified, all multi-item measures were assessed using a 5-point Likert scale ranging from 1 (strongly disagree) to 5 (strongly agree), with higher scores indicating higher levels of the corresponding construct. The study included four multi-item constructs—ability exposure in PE, evaluation anxiety, PE belonging, and psychological well-being—as well as one dummy-coded contextual variable, disadvantaged status in PE. In order to keep the main text concise, only a brief overview of each construct is provided here, while the full indicator system and item content are presented. Ability exposure in PE was conceptualized as the extent to which students perceived their bodily performance, movement competence, and physical shortcomings as publicly visible and open to comparison or judgment during PE classes. This construct was measured with six items adapted to the university PE context, and higher scores indicated a stronger sense of being publicly exposed in PE settings; in the present study, the scale demonstrated good reliability (Cronbach’s *α* = 0.887). Evaluation anxiety referred to students’ tension, worry, and self-consciousness arising from the possibility of being judged by teachers or peers in PE contexts. It was assessed using six items capturing fear of negative evaluation, embarrassment, and performance-related nervousness, with higher scores indicating greater evaluation anxiety; the scale showed strong internal consistency in the current sample (Cronbach’s *α* = 0.902). PE belonging was defined as the extent to which students felt accepted, respected, supported, and socially anchored in PE classes. This construct was also measured with six items reflecting peer acceptance, relational comfort, and sense of membership, and higher scores represented a stronger sense of belonging in PE settings; the reliability of the scale was high (Cronbach’s *α* = 0.906). Psychological well-being was assessed as students’ overall positive emotional functioning and subjective psychological condition in university life. It was measured with six items covering positive affect, emotional stability, self-satisfaction, and vitality, with higher scores indicating better psychological well-being; the scale demonstrated good reliability (Cronbach’s *α* = 0.889). In addition, disadvantaged status in PE was measured with a single self-identification item asking whether the respondent perceived themselves as being in a relatively disadvantaged or unfavorable position in PE contexts, coded as 0 = no and 1 = yes. The item was intended to capture a broad subjective sense of positional vulnerability in PE rather than an administrative, diagnostic, or externally assigned category. Accordingly, it may reflect students’ own experiences of low competence, embarrassment, exclusion, body-related concerns, or other reasons for feeling disadvantaged in PE participation. Perceived athletic competence was not used as a focal contextual condition because it primarily captured self-rated skill level, whereas disadvantaged status was intended to represent this broader experiential self-perception. Accordingly, perceived athletic competence was retained as an analytically important covariate. Several demographic and behavioral variables were further included as control variables to account for their potential associations with the focal constructs, including gender (1 = male, 2 = female), grade (1 = freshman, 2 = sophomore, 3 = junior), major type (1 = arts, 2 = science), perceived athletic competence (1 = very weak to 5 = very strong), and weekly exercise frequency (1 = almost none to 5 = 6 + times per week). Because several measures were adapted to the university PE context, particular attention was paid during instrument preparation to item clarity and contextual appropriateness; however, a formal pre-administration face-validity study was not conducted, which should be considered when interpreting the measurement quality (Allen et al., 2023).

### 3.3 Data analysis

Data analysis proceeded in several stages. Descriptive statistics were first calculated to summarize the demographic characteristics of the sample and the distributional properties of the main study variables. Means, standard deviations, and Pearson correlation coefficients were computed to provide an initial overview of the associations among ability exposure, evaluation anxiety, PE belonging, psychological well-being, and disadvantaged status. The psychometric properties of the scales were then examined from a measurement-model perspective. Internal consistency reliability was assessed using Cronbach’s alpha, convergent validity was evaluated through composite reliability (CR) and average variance extracted (AVE), and discriminant validity was assessed by comparing the square roots of the AVE values with the inter-construct correlations. In addition, a confirmatory factor analysis (CFA) was used to evaluate the hypothesized four-factor measurement structure, and a one-factor model was estimated as a rough supplementary check related to common method bias. Given the regression-based design of the study, scale scores were computed as the means of constituent items and then used in the conditional-process analyses.

The hypothesized direct and indirect effects were subsequently tested using hierarchical regression analyses. Ability exposure was first entered as a predictor of evaluation anxiety and then as a predictor of psychological well-being. Evaluation anxiety was added in a subsequent step to examine whether it statistically mediated the association between ability exposure and psychological well-being. To further assess the robustness of the indirect effect, a bootstrapping procedure with 3,000 resamples was used to estimate the indirect effect and its 95% confidence interval, with significance inferred when the confidence interval did not include zero. Moderation analyses were then conducted as supplementary contextual tests to examine whether PE belonging buffered the association between ability exposure and evaluation anxiety and whether disadvantaged status intensified the association between evaluation anxiety and psychological well-being. Prior to creating interaction terms, continuous predictors were standardized, and interaction terms were computed by multiplying the corresponding standardized variables. Standard errors, 95% confidence intervals, exact *p*-values, and variance inflation factors (VIFs) were inspected when evaluating the regression models. In addition, conditional indirect effects were estimated across combinations of low versus high PE belonging and disadvantaged versus non-disadvantaged status in order to examine the possibility of moderated mediation. Because the study relied on same-source self-report data collected at one time point, the regression results were interpreted conservatively, with attention to effect precision, common method concerns, and the nonsignificant indices of moderated mediation. In the results section, standardized regression coefficients are reported as *β* values, whereas the bootstrapped direct, indirect, and total effects reported in the mediation tables are unstandardized effect estimates; these quantities are conceptually related but should not be interpreted as numerically identical.

## 4 Results

### 4.1 Descriptive statistics and correlations among the study variables

As shown in Table 1, the sample was relatively balanced across gender, grade level, and major type. Female students constituted 52.6% of the sample, while male students accounted for 47.4%. Freshmen, sophomores, and juniors represented 31.3, 35.0, and 33.6% of the participants, respectively. With respect to perceived athletic competence, the largest group rated themselves as average (37.8%), followed by relatively strong (23.3%) and relatively weak (18.5%). A total of 26.7% of students reported being in a disadvantaged position in PE settings, suggesting the presence of a meaningful at-risk subgroup within the sample.

**Table 1:** Demographic characteristics of the participants.

Variable	Category	*n*	%
Gender	Male	307	47.4
Gender	Female	341	52.6
Grade	Freshman	203	31.3
Grade	Sophomore	227	35
Grade	Junior	218	33.6
Major type	Arts	309	47.7
Major type	Science	339	52.3
Perceived athletic competence	Very weak	66	10.2
Perceived athletic competence	Relatively weak	120	18.5
Perceived athletic competence	Average	245	37.8
Perceived athletic competence	Relatively strong	151	23.3
Perceived athletic competence	Very strong	66	10.2
Weekly exercise frequency	Almost none	95	14.7
Weekly exercise frequency	Once per week	123	19
Weekly exercise frequency	2–3 times/week	236	36.4
Weekly exercise frequency	4–5 times/week	131	20.2
Weekly exercise frequency	6 + times/week	63	9.7
Disadvantaged status	No	475	73.3
Disadvantaged status	Yes	173	26.7

Table 2 shows that ability exposure was positively correlated with evaluation anxiety (*r* = 0.579, *p* < 0.001) and negatively correlated with PE belonging (*r* = −0.296, *p* < 0.001) and psychological well-being (*r* = −0.451, *p* < 0.001). Evaluation anxiety was negatively associated with both PE belonging (*r* = −0.486, *p* < 0.001) and psychological well-being (*r* = −0.651, *p* < 0.001). PE belonging, in turn, was positively associated with psychological well-being (*r* = 0.590, *p* < 0.001). Disadvantaged status was positively correlated with ability exposure and evaluation anxiety, but negatively correlated with PE belonging and psychological well-being. Overall, the correlation matrix was consistent with the hypothesized direction of effects.

**Table 2:** Descriptive statistics and bivariate correlations among the study variables.

Variable	M	SD	1	2	3	4	5
Ability exposure	3.36	0.66	—	0.579***	−0.296***	−0.451***	0.202***
Evaluation anxiety	3.28	0.70		—	−0.486***	−0.651***	0.320***
PE belonging	3.62	0.65			—	0.590***	−0.243***
Psychological well-being	3.58	0.61				—	−0.279***
Disadvantaged status	0.27	0.44					—

*** *p* <.001.

### 4.2 Reliability, convergent validity, and discriminant validity of the study variables

As reported in Table 3, all constructs demonstrated satisfactory reliability and convergent validity. Cronbach’s *α* coefficients ranged from 0.887 to 0.906, while composite reliability values ranged from 0.888 to 0.906. In addition, all AVE values exceeded the recommended threshold of 0.50, and the standardized factor loadings ranged from 0.720 to 0.796 for ability exposure, 0.751–0.791 for evaluation anxiety, 0.775–0.796 for PE belonging, and 0.721–0.789 for psychological well-being. The hypothesized four-factor CFA showed an acceptable fit to the data, *χ*^2^(246) = 228.94, *p* = 0.776, CFI = 1.00, TLI = 1.00, RMSEA = 0.00. By contrast, a one-factor model fit the data substantially worse, *χ*^2^(252) = 3115.54, *p* < 0.001, CFI = 0.680, TLI = 0.649, RMSEA = 0.133. Taken together, these results are broadly consistent with the intended distinctiveness of the focal measures, although they should be interpreted as supportive rather than definitive evidence in a same-source survey design.

**Table 3:** Reliability and convergent validity of the measurement scales.

Construct	Items	Cronbach’s *α*	CR	AVE	Standardized loading range
Ability exposure	6	0.887	0.888	0.568	0.720–0.796
Evaluation anxiety	6	0.902	0.902	0.605	0.751–0.791
PE belonging	6	0.906	0.906	0.617	0.775–0.796
Psychological well-being	6	0.889	0.889	0.573	0.721–0.789

As shown in Table 4, the square roots of the AVE values for ability exposure, evaluation anxiety, PE belonging, and psychological well-being were all greater than the correlations among the constructs, supporting discriminant validity. Taken together, these results suggest that the focal variables exhibited acceptable psychometric properties for subsequent analyses.

**Table 4:** Discriminant validity of the latent constructs.

Variable	AE	EA	PB	PWB
AE	0.754	0.579	−0.296	−0.451
EA	0.579	0.778	−0.486	−0.651
PB	−0.296	−0.486	0.785	0.59
PWB	−0.451	−0.651	0.59	0.757

### 4.3 Testing the mediating role of evaluation anxiety

Table 5 shows that ability exposure significantly predicted evaluation anxiety (*β* = 0.495, *p* < 0.001), indicating that greater public exposure in PE was associated with stronger evaluative concern. Ability exposure also significantly and negatively predicted psychological well-being (*β* = −0.342, *p* < 0.001). After evaluation anxiety was added in Model 3, it significantly predicted psychological well-being (*β* = −0.496, *p* < 0.001), while the effect of ability exposure was substantially reduced but remained significant [*β* = −0.097, SE = 0.035, *p* = 0.006, 95% CI (−0.166, −0.028)]. The bootstrapped indirect effect was −0.226 [Boot SE = 0.023, 95% CI (−0.273, −0.185)], which is consistent with a partial statistical mediating role of evaluation anxiety. In addition, perceived athletic competence and weekly exercise frequency were consistently associated with lower anxiety and higher well-being, suggesting that both may serve as protective factors in PE settings. Multicollinearity diagnostics were acceptable, with VIF values ranging from 1.01 to 1.70.

**Table 5:** Hierarchical regression analyses for the mediating role of evaluation anxiety.

Variables	Model 1: evaluation anxiety	Model 2: psychological well-being	Model 3: psychological well-being
Gender	0.028	−0.028	−0.014
Grade	0.024	0.007	0.019
Major type	0.067*	−0.066*	−0.033
Perceived athletic competence	−0.237***	0.309***	0.192***
Weekly exercise frequency	−0.134***	0.183***	0.116***
Ability exposure	0.495***	−0.342***	−0.097**
Evaluation anxiety			−0.496***
*R* ^2^	0.412	0.331	0.476
Δ*R*^2^	—	—	0.145***
*F*	74.889***	52.901***	83.007***

*p <.05; **p <.01; ***p <.001.

As shown in Table 6, the total effect of ability exposure on psychological well-being was significant [Effect = −0.316, 95% CI (−0.378, −0.254)]. The direct effect remained significant after controlling for evaluation anxiety [Effect = −0.089, 95% CI (−0.153, −0.025)]. More importantly, the indirect effect of ability exposure on psychological well-being through evaluation anxiety was significant [Effect = −0.226, 95% CI (−0.273, −0.184)], providing further support for the mediating role of evaluation anxiety.

**Table 6:** Bootstrapping test of the direct and indirect effects of ability exposure on well-being.

Path	Effect	Boot SE	95% Boot CI
Total effect: AE → PWB	−0.316	0.031	[−0.378, −0.254]
Direct effect: AE → PWB	−0.089	0.033	[−0.153, −0.025]
Indirect effect: AE → EA → PWB	−0.226	0.022	[−0.273, −0.184]

### 4.4 Testing the moderating effect of PE belonging

As shown in Table 7, ability exposure significantly predicted higher evaluation anxiety (*β* = 0.445, *p* < 0.001), whereas PE belonging significantly predicted lower evaluation anxiety (*β* = −0.274, *p* < 0.001). However, the interaction between ability exposure and PE belonging was not significant [*β* = −0.047, SE = 0.030, *p* = 0.117, 95% CI (−0.106, 0.012)], and the increase in explained variance was very small (Δ*R*^2^ = 0.002). These results suggest that PE belonging was associated with lower evaluation anxiety overall, but did not significantly buffer the positive relationship between ability exposure and evaluation anxiety in this sample.

**Table 7:** Moderating effect of PE belonging on the relationship between ability exposure and evaluation anxiety.

Variables	Model 1	Model 2
Gender	0.019	0.022
Grade	0.022	0.021
Major type	0.058*	0.060*
Perceived athletic competence	−0.139***	−0.139***
Weekly exercise frequency	−0.092**	−0.093**
Ability exposure (AE)	0.446***	0.445***
PE belonging (PB)	−0.276***	−0.274***
AE × PB		−0.047
R^2^	0.471	0.473
ΔR^2^	—	0.002
F	81.263***	71.575***

*p <.05; **p <.01; ***p <.001.

### 4.5 Testing the moderating effect of disadvantaged status and the moderated mediation model

As shown in Table 8A, evaluation anxiety significantly and negatively predicted psychological well-being (*β* = −0.486, *p* < 0.001), while disadvantaged status also showed a negative main effect (*β* = −0.237, *p* < 0.01). However, the interaction between evaluation anxiety and disadvantaged status was not significant [*β* = −0.069, SE = 0.067, *p* = 0.309, 95% CI (−0.201, 0.063)]. These findings indicate that disadvantaged students tended to report lower psychological well-being overall, but disadvantaged status did not significantly strengthen the negative association between evaluation anxiety and well-being.

**Table 8A:** Moderating effect of disadvantaged status on the relationship between evaluation anxiety and psychological well-being.

Variables	Model 1	Model 2
Gender	−0.017	−0.017
Grade	0.021	0.022
Major type	−0.023	−0.024
Perceived athletic competence	0.214***	0.215***
Weekly exercise frequency	0.124***	0.127***
Evaluation anxiety (EA)	−0.506***	−0.486***
Disadvantaged status (DS)	−0.259***	−0.237**
EA × DS		−0.069
R^2^	0.481	0.482
ΔR^2^	—	0.001
F	84.792***	74.327***

**p <.01; ***p <.001.

Table 8B shows that the indirect effect of ability exposure on psychological well-being through evaluation anxiety was significant across all combinations of PE belonging and disadvantaged status, as all bootstrap confidence intervals excluded zero. Descriptively, the indirect effect was strongest among students with low PE belonging and disadvantaged status (Effect = −0.250), and weakest among students with high PE belonging and no disadvantaged status (Effect = −0.172). However, because the formal indices of moderated mediation were not statistically significant, these subgroup differences should be interpreted as descriptive rather than confirmatory.

**Table 8B:** Conditional indirect effects of ability exposure on psychological well-being across levels of PE belonging and disadvantaged status.

PE belonging	Disadvantaged status	Indirect effect	Boot SE	95% Boot CI
Low (−1 SD)	No	−0.212	0.026	[−0.265, −0.164]
Low (−1 SD)	Yes	−0.25	0.037	[−0.323, −0.181]
High (+1 SD)	No	−0.172	0.024	[−0.221, −0.128]
High (+1 SD)	Yes	−0.202	0.031	[−0.268, −0.145]

As shown in Table 8C, the index of moderated mediation for the path from ability exposure to evaluation anxiety moderated by PE belonging was not significant, as the bootstrap confidence interval included zero. Similarly, the index for the path from evaluation anxiety to psychological well-being moderated by disadvantaged status was also not significant. Therefore, the hypothesized moderated mediation model was not supported, and any conditional-process interpretation should remain cautious.

**Table 8C:** Indices of moderated mediation.

Moderated path	Index	Boot SE	95% Boot CI
AE → EA moderated by PB	0.02	0.013	[−0.005, 0.048]
EA → PWB moderated by DS	−0.034	0.028	[−0.088, 0.021]

Taken together, the results provide strong support for the central mediating role of evaluation anxiety in linking ability exposure to psychological well-being in university PE contexts. Students who experienced greater exposure of their bodily competence tended to report stronger evaluation anxiety, which in turn was associated with poorer psychological well-being. PE belonging and disadvantaged status both showed meaningful main effects, indicating that social inclusion and structural position matter for students’ emotional experiences in PE. However, neither moderator significantly altered the focal structural paths, suggesting that the exposure–anxiety–well-being mechanism may operate relatively broadly across student subgroups. In substantive terms, the findings imply that the psychological costs of being seen and judged in PE are not confined to narrowly defined vulnerable groups, but may reflect a more general feature of evaluative bodily pedagogy.

## 5 Discussion

### 5.1 From being seen to feeling judged: ability exposure as a source of evaluative vulnerability in PE

The present study provides consistent support for the argument that the public visibility of bodily competence in PE is not a neutral pedagogical condition, but a psychologically consequential experience. As shown in Tables 2, 5, ability exposure was positively associated with evaluation anxiety, and evaluation anxiety, in turn, was strongly and negatively related to psychological well-being. This pattern suggests that what matters in PE is not merely whether students participate in physical activity, but whether participation occurs under conditions of public observability, comparison, and potential judgment. In this respect, the present findings are consistent with earlier research showing that PE is not simply a movement-based learning context, but also an emotionally charged setting in which visibility, comparison, and classroom exposure can become sources of stress and discomfort for students (Leisterer and Jekauc, 2019; Tudor et al., 2018; Metz et al., 2024). The findings also extend work suggesting that students’ experiences in PE are shaped by being seen, interpreted, and socially positioned through their bodily performance (Åsebø et al., 2021; Andresen et al., 2023). However, whereas much of this prior research has tended to document emotional experience, discomfort, or classroom stress more descriptively, the present study goes further by specifying a clearer explanatory mechanism through which such experiences are translated into diminished psychological well-being. In such contexts, the body becomes socially readable, and movement performance becomes a basis for self-evaluation and anticipated peer evaluation. For many students, especially those who do not perceive themselves as athletically competent, PE may therefore function less as a health-promoting setting and more as a site of heightened evaluative pressure, a conclusion that is also in line with earlier evidence linking perceived athletic competence to fear of negative evaluation in PE settings (Ridgers et al., 2007).

The mediation results help clarify how ability exposure may be linked to lower well-being in PE. Tables 5, 6 show that ability exposure was associated with poorer psychological well-being both directly and indirectly through evaluation anxiety. In practical terms, this suggests that visibility in PE may become problematic when students interpret it as a cue for possible judgment. This reading is consistent with work on social-evaluative threat (Dickerson and Kemeny, 2004) and with research showing that anticipated evaluation can heighten self-consciousness and withdrawal (Morrison and Heimberg, 2013; Weeks and Howell, 2012). The contribution of the present study is to locate this process in the specific setting of university PE, where performance is immediate, public, and hard to conceal. The findings therefore support H1, H2, and H3 while also suggesting that some routine pedagogical practices, such as public demonstrations and visible comparison, may carry psychological costs for students.

### 5.2 Belonging and disadvantage as background conditions: protective and risk factors without strong boundary effects

The findings also show that social position and classroom belonging matter, although not in a strongly moderating way. As shown in Tables 2, 7, 8A, PE belonging was associated with lower evaluation anxiety and higher psychological well-being, whereas disadvantaged status was associated with higher anxiety and lower well-being. These patterns are important because they suggest that students enter PE with different levels of relational safety and vulnerability. The results are in line with broader research identifying belonging as a psychosocial resource linked to adjustment and well-being (Allen et al., 2018; Furrer and Skinner, 2003), and with PE scholarship emphasizing the importance of social acceptance and inclusion in movement-based settings (O'Neil and Olson, 2021). They also fit recent evidence from higher education showing that belonging is closely tied to students’ well-being and academic experience (Galioto et al., 2025). At the same time, the pattern for disadvantaged status is consistent with inclusive PE research showing that less advantaged students often report lower confidence and a weaker sense of safety in participation (Goodwin and Watkinson, 2000; Block and Obrusnikova, 2007; Bertills et al., 2018).

However, the moderation analyses did not provide strong support for the claim that PE belonging or disadvantaged status significantly altered the focal structural paths. Specifically, the interaction between ability exposure and PE belonging was not statistically significant, and the interaction between evaluation anxiety and disadvantaged status was likewise nonsignificant. The indices of moderated mediation also included zero. At first glance, these findings may seem inconsistent with a strong conditional-process account. Yet a more careful interpretation suggests something theoretically meaningful: belonging and disadvantage may operate less as boundary conditions that change the slope of the core mechanism, and more as background conditions that shape students’ overall emotional climate. In other words, PE belonging appears to function as a general protective resource, whereas disadvantaged status functions as a general risk factor, but neither was sufficient in this study to fundamentally transform the exposure–anxiety–well-being sequence. This reading also helps refine existing inclusion-oriented scholarship. Prior research has often emphasized that inclusion, acceptance, and positional vulnerability matter for students’ experiences in PE, but has been less explicit about whether such factors reshape the internal mechanism through which evaluative exposure affects well-being, or whether they instead influence the broader conditions under which that mechanism operates. The present findings lend stronger support to the latter interpretation. This is also consistent with the pattern of conditional indirect effects reported in Table 8B. Descriptively, the indirect effect was stronger among students with low belonging and disadvantaged status, and weaker among those with high belonging and no disadvantaged status. The direction of these differences aligns with theory, but the formal tests indicate that they should be interpreted cautiously. This combination of directional consistency and statistical nonsignificance suggests that the core mechanism may be relatively robust across groups, even if some students are generally better or worse positioned within the PE context. Such a result is not trivial. On the contrary, it implies that the psychological costs of evaluative bodily exposure may extend beyond narrowly defined vulnerable subgroups and may reflect a more general feature of performance-visible PE environments. Thus, H4 was supported at the level of main effects, whereas H5 received only partial support. Rather than weakening the study, this pattern helps clarify that social belonging and disadvantage shape the baseline emotional conditions under which students encounter PE, while the central exposure–anxiety–well-being pathway remains comparatively stable across subgroups. Put differently, inclusion-related resources and vulnerabilities matter, but they may do so by influencing students’ general sense of safety, acceptance, and psychological readiness rather than by sharply altering the structural logic through which exposure translates into anxiety and reduced well-being. This helps move the discussion beyond a narrow moderator-centered interpretation and toward a broader understanding of PE as a relational and stratified emotional environment in which some students begin from more protected positions and others from more fragile ones, even though the fundamental psychological burden associated with public bodily evaluation may be shared more widely than initially assumed.

### 5.3 Rethinking inclusive PE: from subgroup sensitivity to universal emotional design

Taken together, the findings point to a broader view of inclusive PE. The main implication is not only that some subgroups are at risk, but also that evaluative features of PE may be stressful for a wider range of students. Table 1 shows that a substantial minority of participants perceived themselves as disadvantaged, yet the mediation results suggest that the exposure-anxiety-well-being process was not limited to that group alone. This shifts the discussion from who is included to how participation is organized. Existing work has shown that social acceptance, belonging, and participation are central to students’ PE experiences (O'Neil and Olson, 2021; Ruscitti et al., 2017), and broader educational research similarly shows that belonging matters for adjustment and well-being (Allen et al., 2018; Walton and Cohen, 2011). The present findings add that the emotional design of participation also deserves attention. If being watched, compared, and assessed in embodied ways reliably coincides with higher anxiety, then inclusion in PE should involve not only access, but also classroom conditions that make participation feel less threatening.

From this perspective, the study contributes to the literature by linking inclusion, mental health, and classroom experience through a concrete psychological mechanism. Rather than assuming that PE is inherently beneficial because it involves movement, the present findings suggest that the emotional meaning of movement matters critically. This conclusion helps make sense of why PE research has often produced mixed accounts, with some studies emphasizing the developmental, social, and emotional benefits of participation, while others highlight discomfort, stress, or vulnerability in visible performance situations (Leisterer and Jekauc, 2019; Metz et al., 2024). The current study helps reconcile these strands by suggesting that PE can support well-being when students experience competence, acceptance, and safety, but can undermine well-being when bodily participation is structured around visibility and judgment. In this sense, the study advances a more differentiated account in which mental-health outcomes in PE depend not only on activity itself, but also on whether that activity is experienced as supportive or exposing. The practical implications follow directly from this argument. If evaluation anxiety is the central pathway linking ability exposure to reduced psychological well-being, then PE teachers and curriculum designers should pay closer attention to how performance situations are staged. Instructional strategies that rely heavily on public demonstration, visible ranking, spontaneous individual display, or peer-observable error correction may unintentionally intensify students’ evaluative burden. By contrast, practices that normalize skill diversity, reduce unnecessary public comparison, and strengthen interpersonal safety may help preserve the developmental aims of PE without reproducing avoidable emotional harm. In this sense, the study suggests that psychologically inclusive PE is not merely about accommodating the weakest students; it is about designing learning environments in which bodily participation is less threatening for everyone. Seen in this way, the contribution of the present study lies not only in identifying who may struggle in PE, but in showing how commonplace teaching arrangements can systematically organize emotional risk across the classroom. This broader perspective invites scholars and practitioners to move beyond a remedial approach focused only on visibly vulnerable students and toward a preventive model in which the pedagogical design of visibility, comparison, feedback, and participation is itself treated as a mental-health concern. Such a shift is especially important in university PE contexts, where students may already be negotiating identity, competence, and peer recognition in intensified ways, making the emotional tone of performance situations particularly consequential for their sense of well-being.

## 6 Conclusion and limitations

Taken together, the present study advances a more differentiated understanding of physical education (PE) as not only a developmental and health-promoting context, but also a socially evaluative and psychologically consequential environment. The findings suggest that the emotional cost of PE lies not merely in participation itself, but in participation under conditions in which bodily competence is made publicly visible, socially comparable, and potentially open to judgment. More specifically, the study shows that ability exposure is associated with higher evaluation anxiety, that evaluation anxiety is associated with lower psychological well-being, and that evaluation anxiety serves as a central statistical mechanism linking public bodily exposure in PE to reduced well-being among university students. The findings also indicate that PE belonging and self-perceived disadvantaged status matter in meaningful ways, although not as strong moderators of the focal structural paths. Rather, they appear to function more as broader protective and risk contexts that shape the emotional conditions under which students encounter PE. Accordingly, the present study extends inclusion-oriented research by shifting attention from whether students participate to the emotional conditions under which participation occurs, and by showing that psychologically inclusive PE is not simply a matter of ensuring access, but of designing classroom environments in which bodily participation is less threatening, less judgment-laden, and more emotionally sustainable for a wider range of students.

These conclusions should be interpreted alongside several limitations that define the scope of the present findings. First, because the study relied on a cross-sectional self-report design, the results are best understood as theoretically coherent associations rather than definitive causal effects; future research would benefit from longitudinal, experimental, or classroom observational approaches that clarify temporal order more rigorously. Second, the focal constructs were measured primarily through students’ subjective perceptions, which is appropriate given the study’s concern with lived emotional experience, yet this also leaves room for response bias, social desirability bias, and other same-source distortions. Reports of ability exposure in PE may be particularly sensitive in this regard because visibility can be interpreted differently across students and tasks. Third, the study did not retain the full class enrollment frame, so the precise participation rate could not be reconstructed retrospectively; this should be improved in future survey administration. Fourth, although attention was paid to contextual clarity in the adapted items, a formal face-validity study was not conducted before full data collection (Allen et al., 2023). Fifth, the meaning of visibility in PE is not necessarily uniform. Under some conditions, the presence of others may facilitate performance on well-learned or relatively simple tasks, whereas the same visibility may intensify pressure on difficult or identity-relevant tasks. The present study could not disentangle such social-facilitation dynamics from more threatening forms of evaluation, so future work should examine task difficulty, performance familiarity, classroom climate, and assessment practices more explicitly. Sixth, although the four-factor measurement model was more consistent with the data than the one-factor alternative, these analyses provide only limited reassurance regarding common method bias and do not rule out shared-method inflation in a same-source design. Finally, although PE belonging and disadvantaged status showed meaningful main effects, the hypothesized moderation and moderated mediation effects were not strongly supported. This pattern may indicate that the core mechanism is comparatively robust across student groups, but it may also suggest that these contextual conditions operate in more diffuse ways than captured by the present model.

## Data Availability

The raw data supporting the conclusions of this article will be made available by the authors, without undue reservation.
